# Population evacuation path optimization based on potential field ant colony and extended cellular automata

**DOI:** 10.1371/journal.pone.0314803

**Published:** 2024-12-06

**Authors:** Tiechao Liu, Chao Sun, Ning Sui, Mingxin Shen

**Affiliations:** School of Measurement and Communication Engineering, Harbin University of Science and Technology, Harbin, China; University of Catania, ITALY

## Abstract

An effective safety evacuation program is an important basis for safeguarding the lives of people, and reasonable planning of evacuation routes is of great significance for formulating personnel evacuation plans. This article considers the global search ability of the ant colony algorithm and the local search ability of the artificial potential field. The artificial potential field is integrated into the ant colony algorithm, and combined with the extended Moore type cellular automata, an extended cellular automata model based on the potential field ant colony algorithm is proposed to optimize the calculation of personnel evacuation and path planning in the evacuation area. Analyze the performance of different algorithms in planning path smoothness, total path length, and calculation time from the same location in single exit and multi exit evacuation areas. And to verify the effectiveness of the algorithm, we use part of a teaching building as an evacuation scenario. The results show that combining the potential field ant colony algorithm with the extended Moore type cellular automata for path planning can reduce the number of invalid nodes and redundant turning points in the shortest path, improve the smoothness of the path, improve planning efficiency, and provide a design basis for emergency evacuation of buildings.

## 1. Introduction

With increased group activities and frequent accidents, crowded places often have more significant potential for danger. Evacuation from dangerous places is paramount to ensure the colony’s safety. The key to emergency evacuation is to clear the situation of buildings and create an emergency evacuation plan before the accident. Then, evacuation according to the emergency evacuation plan is done to reduce casualties and property losses effectively. An effective evacuation plan is an essential basis to ensure the safety of people in the building, and the reasonable planning of evacuation routes is of great significance to establishing an evacuation plan.

Cellular Automata is a self-organizing structure with a set of cells. The Cellular Automata (CA) model is a commonly used simulation model for human mobility characteristics [1]. Path planning refers to the search of a mobile robot for a collision-free path from a starting position to a goal position in a static or dynamic workspace containing obstacles according to evaluation criteria such as shorter time or closer distance. Ant Colony Optimization (ACO) was first proposed by Marco Dorigo [2] in 1992, inspired by Ant foraging. Zhang Peihong et al. [3] used an adaptive ant colony algorithm to optimize the evacuation path according to the characteristics of pedestrians under building fire; Mao Xinghua et al. [4] used the ant colony algorithm as a distributed computing, ACO is used to solve the road emergency evacuation strategy selection model because of its strong ability to solve complex problems, easy to implement and simple to calculate. In 2020, Jia Jinzhang [5] and others used a genetic algorithm to deal with the initial pheromone of the ant colony algorithm, using a genetic-ant colony algorithm planning fire evacuation path to improve escape efficiency. In 1986, O. Khaitib [6] first proposed the Artificial Potential Field (APF) to optimize the obstacle avoidance path of the planning robot. Artificial Potential Field is a planning method that simulates the distribution of electric potential field, which is a model used to describe the characteristic structure of the pedestrian movement space, and the negative gradient direction of the potential field is used to complete the path planning [7]. In 2020, Wang Peiliang et al. [8] established a hexagonal cellular automata model, used the potential field to optimize the heuristic function of the ant colony algorithm, and used the particle swarm optimization algorithm to solve the parameters, reducing the search time of the algorithm. In 2023, Dhouib et al. [9] developed an innovative best method, DM-SPP, to solve the standard shortest path problem through three variants: single pair, single source and single target path problems, and applied this method to solve the path planning problem of autonomous mobile robots [10]. In 2024, Dhouib et al. [11] improved the Dhouib-Matrix-SPP (DM-SPP) method in terms of convergence speed and shortest path acquisition, further improving the path finding efficiency of the robot.

In this paper, a human evacuation model based on Cellular Automata (CA) and Extended Moore Cellular Automata (ECA) is proposed, which fuses artificial potential field with Improved Ant Colony Optimization (IACO), and plans evacuation routes for personnel. This paper analyzes the performance of different algorithm planning paths in single-exit and multi-exit evacuation areas, starting from the same location. Through the path planning of some scenarios of a teaching building, the algorithm’s applicability is further verified, and effective guidance for the evacuation of personnel is provided.

## 2. Expand Moore-type cellular automata model

### 2.1. Evacuation of space

**Fig 1:**
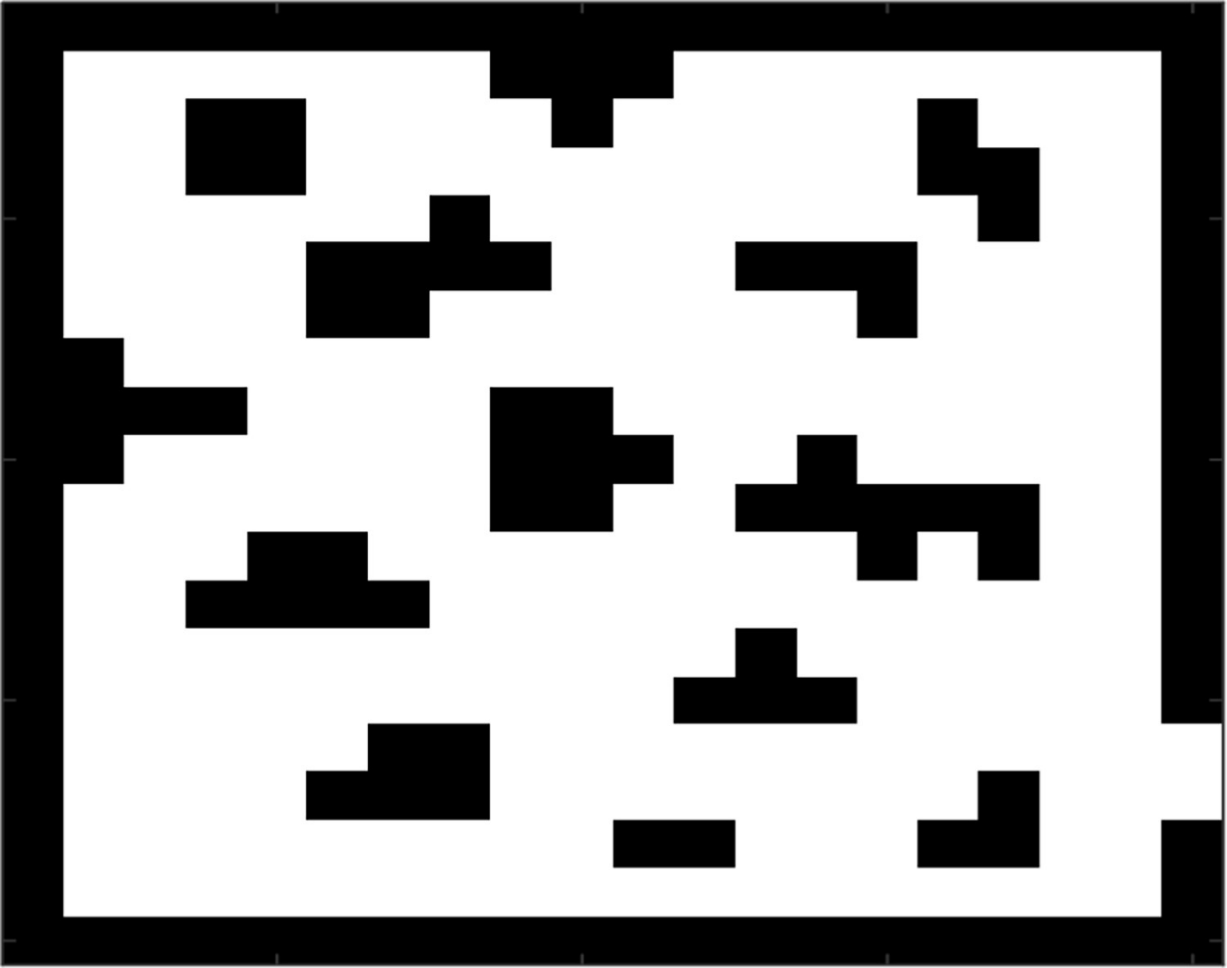
Model diagram.

To better simulate the position of dense colonies in space, the commonly used sizes of each cell in cellular automata models are 0.40m×0.40m and 0.50m×0.50m. In this paper, the evacuation area is discretized into a square with the same size, and the cell space size is 0.40m×0.40m. In cellular space, each cell may have three states: idle, occupied by people, and occupied by obstacles. Each state is assigned a value, and each cell can only have one state simultaneously. The cell state of the obstacle does not change, and the cell state of the person or the idle cell changes with time. In this paper, obstacles less than the size of a cell will be set as a cell to facilitate the study. As shown in Fig 1, obstacles are randomly distributed in the evacuation area. White cells are free cells, and people can pass through; obstacles occupy black cells; people are forbidden to pass through, arrive at the exit, and the evacuation is over.

### 2.2 Expand Moore-type cellular automata

**Fig 2:**
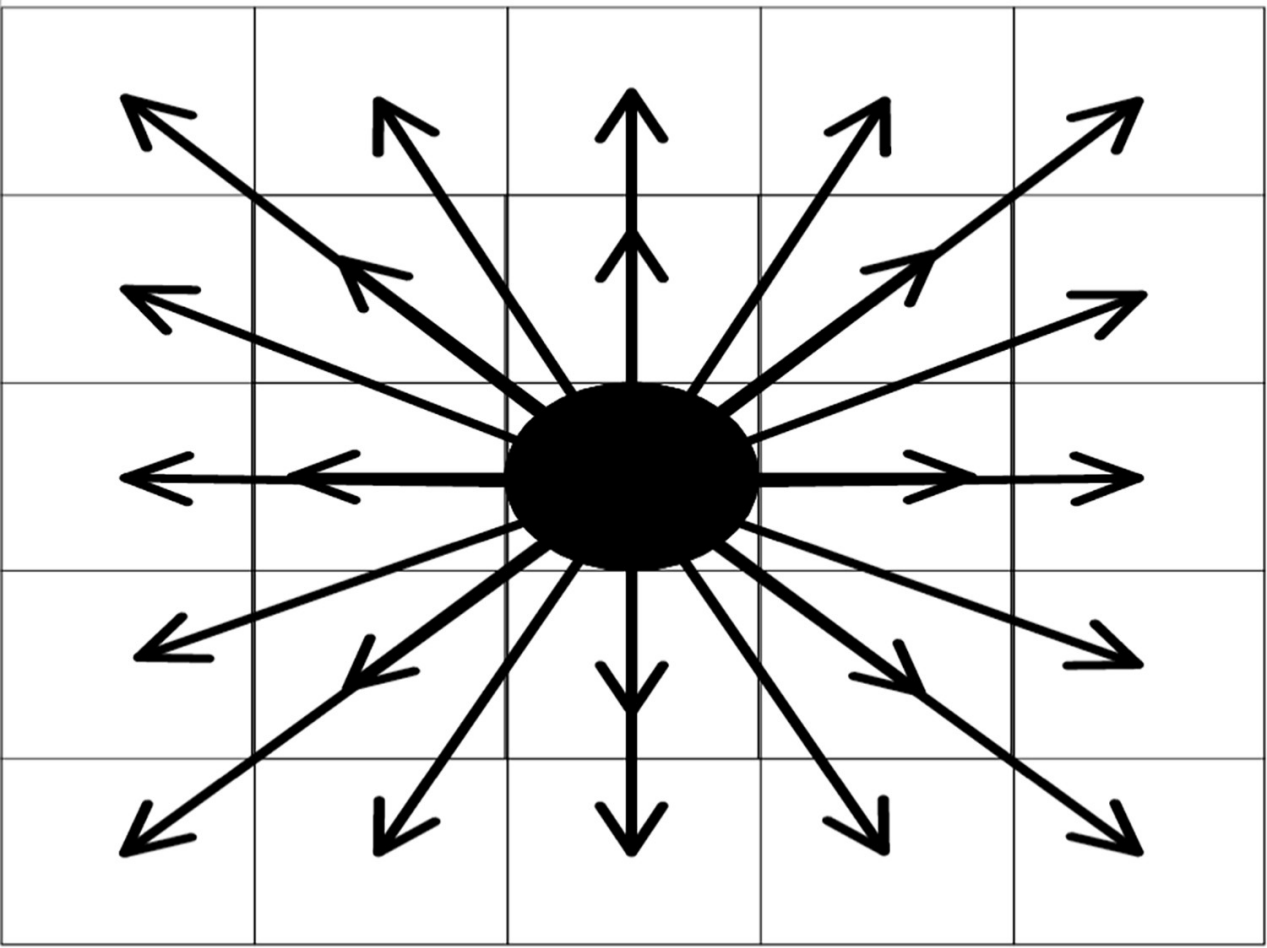
Expand the Moore-type direction of movement.

In this paper, based on the actual situation of evacuation, to speed up the search for the next node in the process of people moving, an extended Moore-type neighborhood is adopted. Fig 2 is an extended Moore-type cellular automaton motion direction indicator; the center cell has 24 motion directions, and the angular resolution of the cell is 22.5°. The center cell can read the state of the 24 surrounding cells, combine the state itself, and import the state information into the movement rule, then calculate the movement probability of the center cell to the surrounding 24 expanding cells. The resolution of 22.5° allows each cell to be more nuanced in its perception of its surroundings, which helps to more precisely capture subtle changes in the evacuation path. The 24 directions provide more movement options, and people may not just move in strict directions (e.g., north, south, east, west). The 24 directions simulate this natural behavior more realistically.

The mathematical definition of an extended Moore-type neighborhood is shown in Formula (1):

$$N_{moore}=\{v_{i}=(v_{ix},v_{iy})\left||v_{ix}-v_{ox}|+|v_{iy}+v_{oy}||\right\}\leq 2,(v_{ix},v_{iy}\in Z)$$ (1)

In the formula, *v*_*ix*_ and *v*_*iy*_ represent the row and column coordinates of the expanded neighbor, and *v*_*ox*_ and *v*_*oy*_ represent the row and column coordinates of the central cell.

The individual’s next step state is determined by its state and the state of neighboring cells, as shown in Formula (2). When multiple people move simultaneously to the same free cell, they randomly select a person to enter the free cell and others in the original position to search for other mobile cells.


$$S_{i}^{t+1}=f(S_{i}^{t},S_{N}^{t})$$ (2)

In formula: $S_{i}^{t}$ represents the cellular state in the center of Time T, and $S_{N}^{t}$ represents the combination of cellular states in the neighborhood of Time T.

### 2.3 Characteristics of human behavior

When an emergency occurs, the evacuation of personnel is affected not only by the distance, exit location, exit width, and other objective factors but also by personal psychological and behavioral factors [12]. Mental and behavioral parameters are given to all people, including colony psychology [13] and mindset [14]. Each state of mind has different behavior patterns, which can be divided into herd behavior and habitual behavior.

The herd effect, also known as the“Herd effect” [15] refers to individuals who ignore their views and judgment and make the same decisions as the surrounding group. Under the influence of colony psychology, people will follow most people around them to make a herd behavior. In terms of evacuation, appropriate herd behavior can enable people unfamiliar with the site to find the exit quickly, thus benefiting the overall evacuation process. Still, when the colony rate is too high, it will increase the evacuation pressure of an exit, which is not conducive to evacuating people, thus having a negative impact.

## 3 Potential field ant colony cellular automata model

### 3.1 Heuristic function and transition probability

**Fig 3:**
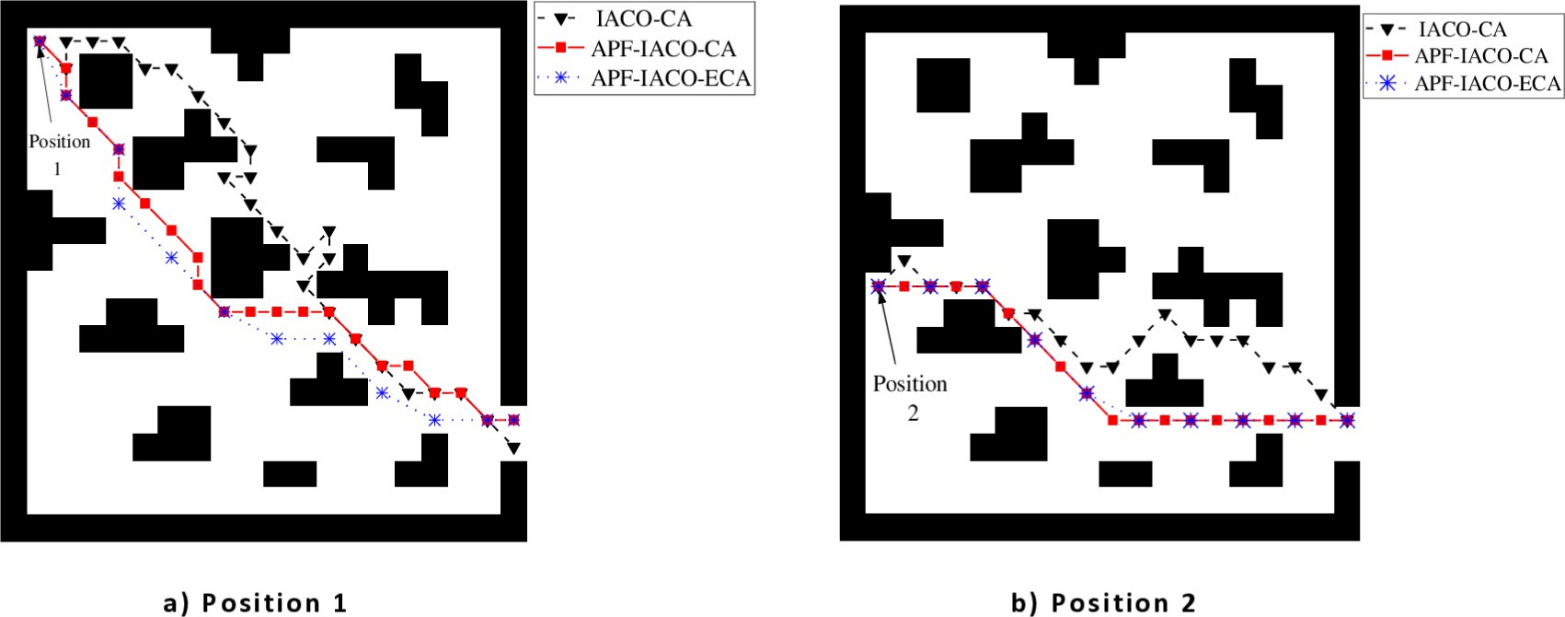
Personnel movement path.

The heuristic function in the traditional ant colony algorithm is usually the reciprocal of the distance between nodes. In contrast, the distance between adjacent nodes in the CA model is a cons. Hence, the value of the heuristic function in the CA model is constant and not inspiring. The risk degree reflects the distance from the candidate node to the exit, and the closer the exit is, the smaller the value is. In this paper, the Breadth First Search (BFS) method was used to calculate the Breadth-First Search of each cell to the exit. As shown in Fig 3, the transition probability is shown in Formula (3)

$$P_{ij}^{k}(t)=\{\begin{array}{cc} \frac{{\left[\tau_{ij}(t)\right]}^{\alpha }\times {\left[\eta_{ij}(t)\right]}^{\beta }}{\sum_{s\in j_{i}}{\left[\tau_{is}(t)\right]}^{\alpha }\times {\left[n_{is}(t)\right]}^{\beta }}, & \mathrm{if}\;j\in J_{i}\\ 0, & \mathrm{Other} \end{array}$$ (3)

In the formula, *J*_*i*_ is a selectable set of cells in the neighborhood, *α、β* are the influence factors of the pheromone *τ*_*ij*_*(t)* and the heuristic function *η*_*ij*_*(t)*, respectively. It reflects the relative importance of information and heuristic information in people’s decision to choose their path.

### 3.2 Pheromone update and contraindication rules

Path planning can be divided into global and local [16, 17]. Ant colony algorithm is usually divided into three models -—the ant-perimeter model, the ant-quantity model, and the ant-density model [18]. In this paper, the pheromone concentration function reflects the updating mode of pheromone using the ant-cycle model. After completing a cycle, all staff update pheromone according to Formula (4) and Formula (5):

$$\tau_{ij}(t+n)=(1-\rho )\times \tau_{ij}(t)+\Delta \tau_{ij}(t)$$ (4)

$$\Delta \tau_{ij}(t)=\sum_{k=1}^{m}\Delta \tau_{ij}^{k}(t)$$ (5)

In the formula, *ρ* is the volatilization coefficient, *τ*_*ij*_*(t)* is the amount of pheromone on the t-moment path (*i*, *j*). The amount of pheromone on the path (*i*, *j*) that the person k increases during the period N represents the number of times that person K passed the path (*i*, *j*) during this walk. See Formula (6)

$$\Delta \tau_{ij}^{k}(t)=\{\begin{array}{l} \frac{1}{L_{k}}\times {\left(\frac{1}{2}\right)}^{n-1},\;\mathrm{The}\;\mathrm{person}\;\mathrm{passes}\;\mathrm{through}\;(i,j)\;\mathrm{in}\;k\;\mathrm{cycles}\\ \;0,\;\mathrm{Other} \end{array}$$ (6)

In the formula: $\Delta \tau_{ij}^{k}(t)$ is *t*+*Δt* is the number of pheromones on the b-moment path (i, J), and *L*_*k*_ is the number of cells that person K passes through in this traversal.

In the cellular automata model based on the ant colony algorithm, one ant is one person:

1. A cell can only be occupied by one person, and people can only enter cells occupied by idle cells, obstacles, or other people; people can not enter.
2. When many people occupy the same free cell simultaneously, one person is chosen to enter the free cell at random, and those who can not enter the free cell continue to search for other suitable free cells.
3. A person can not select a cell that has passed unless no other cell can be accessed.

### 3.3 Artificial potential field

The artificial potential field method abstractly represents the robot’s working space as a virtual potential field. The target point generates a gravitational field, the obstacle generates a repulsive field, and the two superimpose to form a resultant force field [19, 20]. The ant colony optimization algorithm and artificial potential field are combined to make up for the deficiency of the two algorithms. The application of artificial potential field to ant colony algorithm can make up for the shortcomings of ant colony algorithm in local search and convergence speed, and help the algorithm jump out of local optimum through the deterministic guidance of potential field, and quickly adapt in the dynamic environment, so as to improve the overall search efficiency and quality of solution. In the extended cellular automaton evacuation model, each evacuator is an ant. The gravitational and repulsive potential energy of member *k* at time *t* are defined as Formulas (7) and (8), respectively.


$$U_{att}(i)=\frac{1}{2}\xi \rho_{g}^{2}(i)$$ (7)

$$U_{rep}(i)=\{\begin{array}{cc} \frac{1}{2}\gamma (\frac{1}{\rho (i)}-\frac{1}{\rho_{0}}), & \rho (i)\leq \rho_{0}\\ 0, & \rho (i)>\rho_{0} \end{array}$$ (8)

In the formula, *ρ_g_*(*i*) is the Euclidean distance from node i to the target, *ξ* is the attractive potential energy factor, *γ* is the repulsive potential energy factor, and *ρ(i)* is the shortest distance between person *k* and obstacle *ρ*_*0*_ is the maximum distance that an obstacle affects a person.

## 4 Comparison and analysis of simulation results

The path planning performance of the improved ant colony cellular automata model (IACO-CA), potential field ant colony algorithm cellular automata model (APF-IACO-CA), and potential field ant colony algorithm extended Moore Cellular Automata Model (APF-IACO-ECA) are compared to prove the validity of this model in path planning. The result of LYU Chen [21] study shows that the critical density of people in a large building is 0.7 people/m^2^, the movable area of people in the evacuation area is 42 m^2^, which can accommodate up to 25 people, in the evacuation model, 25 people were randomly distributed.

### 4.1 Comparison of different model paths at the same location

25 people were randomly distributed in the evacuation area, randomly assigned to different mental states, using the above three models were tested. In the experiment, the person’s position and mental state were the same thrice. Set the initial position of the upper left corner to position 1 and the middle left corner to position 2. The paths planned using three different algorithms are shown in Fig 3.

As shown in Fig 3, all three algorithms can plan the path to the exit, and IACO-CA is not the optimal path, which is quite different from the other two algorithms, APF-IACO-CA and APF-IACO-ECA are relatively close to each other. When the initial position is in the upper left corner, the path of the APF-IACO-CA algorithm and the APF-IACO-ECA algorithm has less overlap than the path of the middle position on the left side, APF-IACO-ECA can better reflect the advantages of the algorithm.

#### 4.1.1. Path smoothness analysis

Table 1 shows that when the initial position is 1, the IACO-CA algorithm has the most turns and planning times, APF-IACO-CA is the next, and APF-IACO-ECA is the least. The number of turns in APF-IACO-CA is 1 and 4 times less than in IACO-CA. APF-IACO-CA has the smoothest path than IACO-CA, which reduces the number of turns by 6 times and the number of turns by 14 times. When the initial position is position 2, the APF-IACO-CA has the least number of turns, and the APF-IACO-ECA has the least number of turns. Therefore, the further away the initial position is from the exit, the better the advantage of the APF-IACO-ECA algorithm is.

**Table 1:** Path comparison.

Algorithm	IACO-CA		APF-IACO-CA		APF-IACO-ECA	
Position	1	2	1	2	1	2
Number of turns/time	15	13	14	3	9	4
Number of planning times/time	25	18	21	18	11	9

#### 4.1.2. Planning total path length analysis

**Fig 4:**
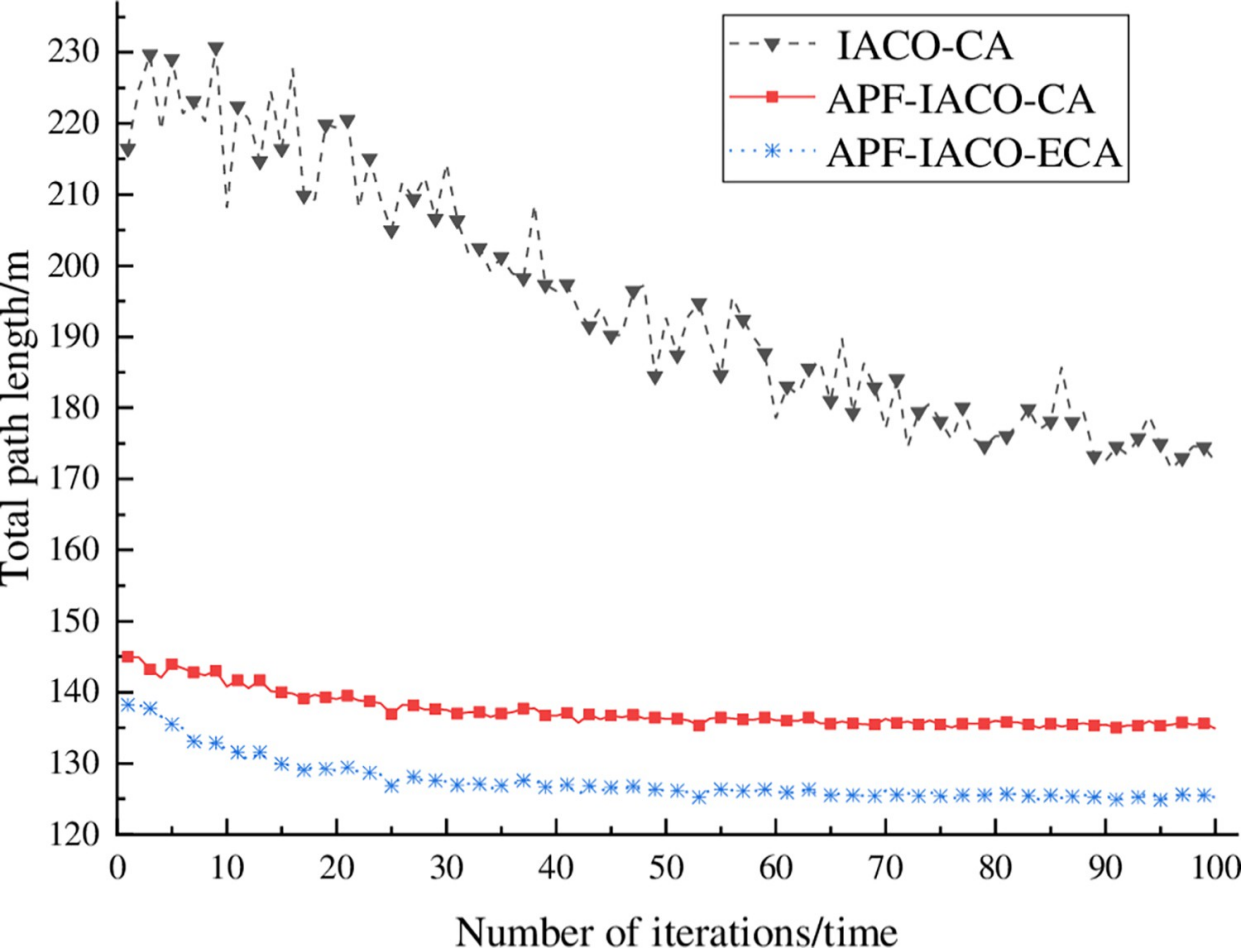
Plan the total path length curve.

Fig 4 shows the curve of the total path length of 25 entirely evacuated people with the number of iterations. The total path length of IACO-CA fluctuates wildly, but the overall trend is that the total path length decreases with the number of iterations. According to Table 2, the shortest total path length of APF-IACO-CA is 134.91 m. The shortest total path length of APF-IACO-ECA is 125.21 m, and the total path length is shortened by 7.75%.

**Table 2:** Comparison of path distances.

algorithm	IACO-CA	APF-IACO-CA	APF-IACO-ECA
distance/m	172.40	134.91	125.21

#### 4.1.3. Planning total calculation time analysis

**Fig 5:**
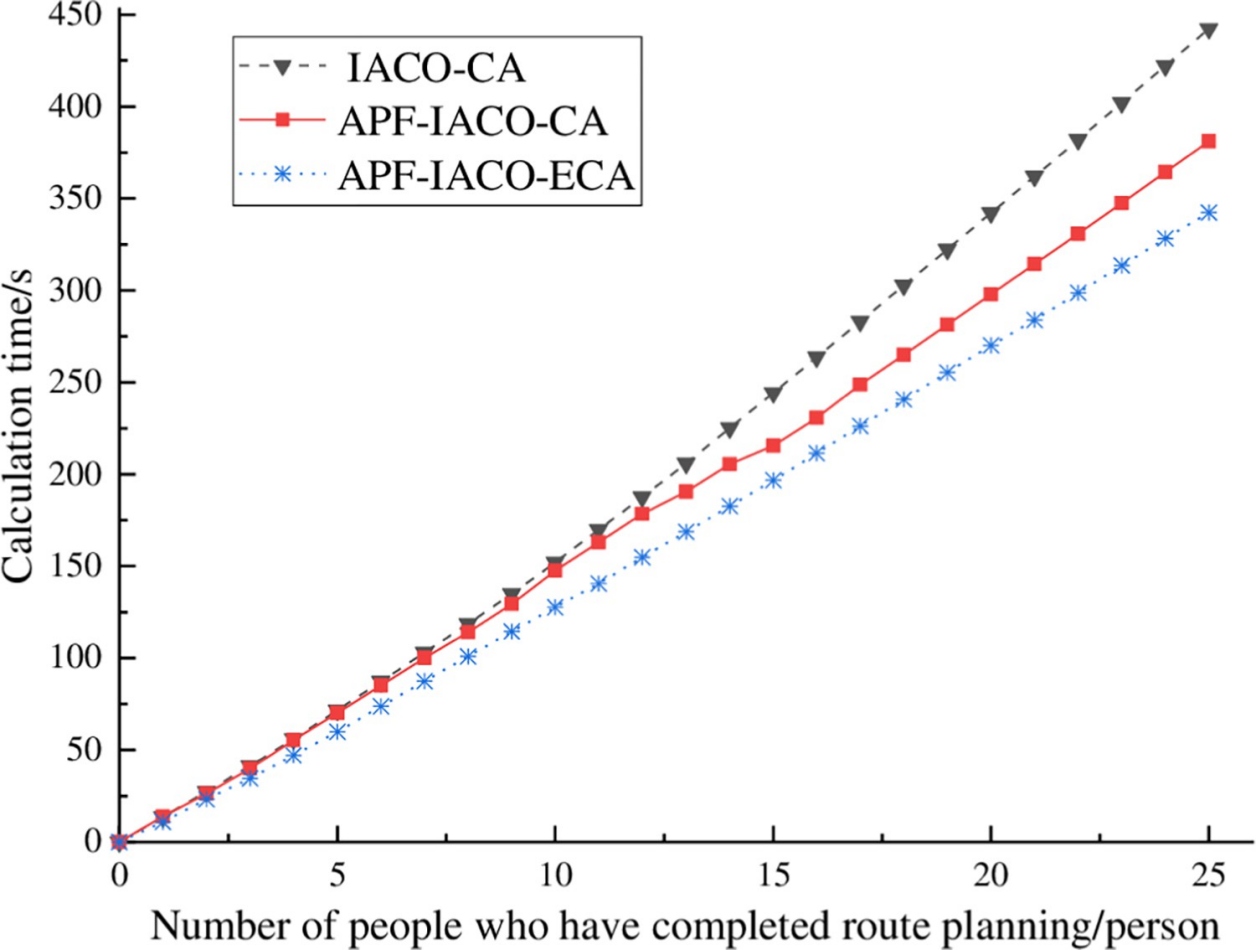
Total calculation time curves.

Fig 5 and Table 3 respectively show the curves and statistical tables of the number of people who completed the path planning and the calculation time. It takes 442.40 s to complete the path planning using the IACO-CA algorithm and less computation time using the APF-IACO-CA algorithm and the APF-IACO-ECA algorithm, which requires 381.13 s and 342.26 s, respectively. The APF-IACO-ECA algorithm saves 61.27 s compared with the IACO-CA algorithm, and the APF-IACO-ECA algorithm saves 38.87 s compared with the APF-IACO-CA algorithm. Therefore, the APF-IACO-ECA algorithm takes the least time to plan the path and computes the fastest. When the path planning is combined with the actual situation, the optimal path planning can be completed more effectively, proving the algorithm’s effectiveness.

**Table 3:** Comparison of calculation time.

algorithm	IACO-CA	APF-IACO-CA	APF-IACO-ECA
time/s	442.40	381.13s	342.26s

### 4.2 Dual exit area path comparison

In the building design fire code, there is more emphasis on the number of building safety exits. To prevent emergencies, to speed up evacuation, and to avoid one exit not working, a single room often has more than one exit. The analysis of double exits on the same side of the wall at both ends shows that the double exits’ width is equal.

**Fig 6:**
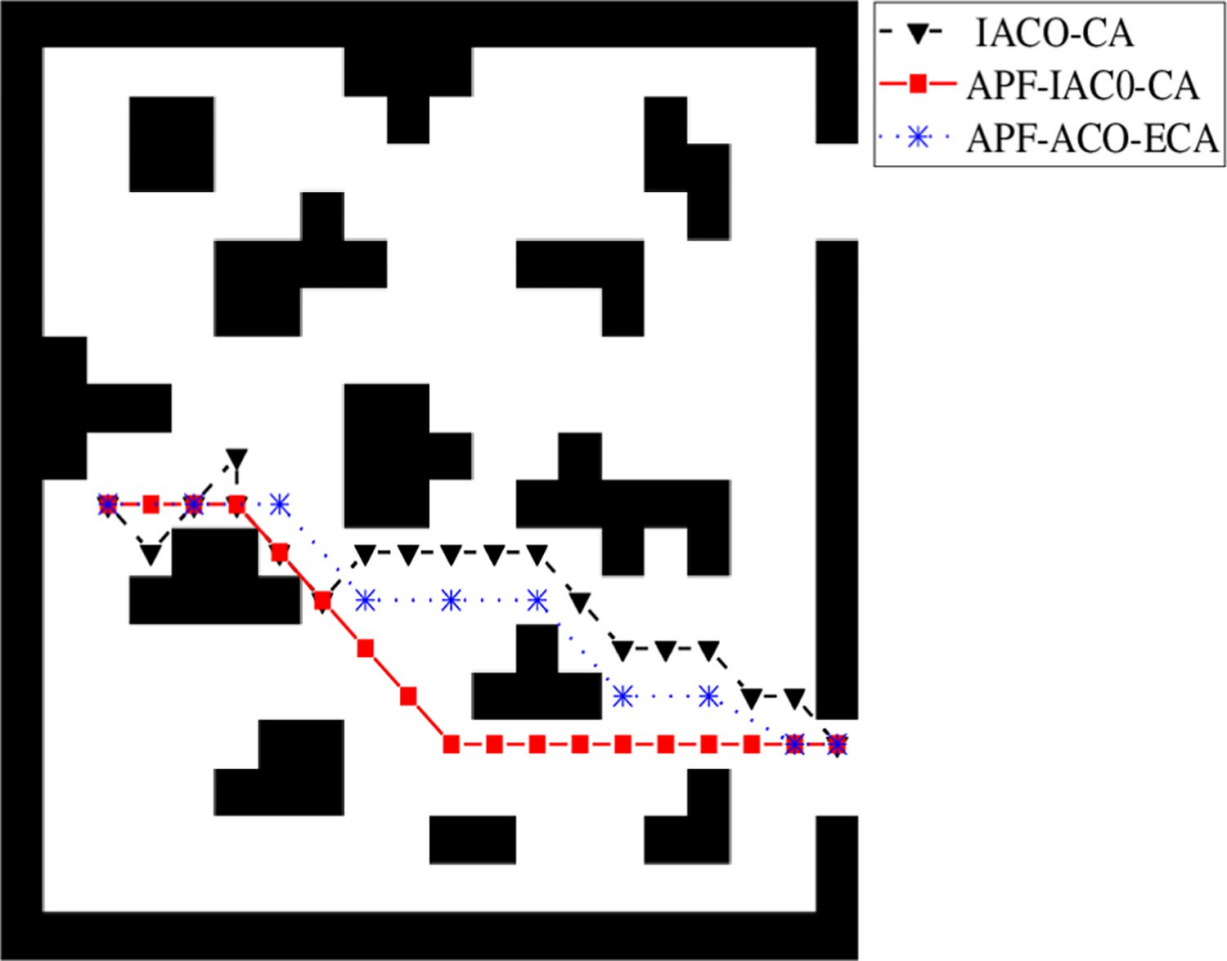
Dual exit path plan.

Fig 6 is the path map of the three algorithms in a certain location of the dual-outlet area. All three algorithms can plan the path to the nearest exit, but the direction choice differs significantly.

#### 4.2.1. Path smoothness analysis

As can be seen from Table 4, the IACO-CA algorithm has the most number of turns and planning times. APF-IACO-CA has the least number of turns, and APF-IACO-ECA has the least. The planning efficiency of APF-IACO-ECA is 57.89% higher than that of APF-IACO-CA.

**Table 4:** Path comparison.

Algorithm	IACO-CA	APF-IACO-CA	APF-IACO-ECA
Number of turns/turns	11	3	7
Planning frequency/frequency	19	18	8

#### 4.2.2. Planning total path length analysis

**Fig 7:**
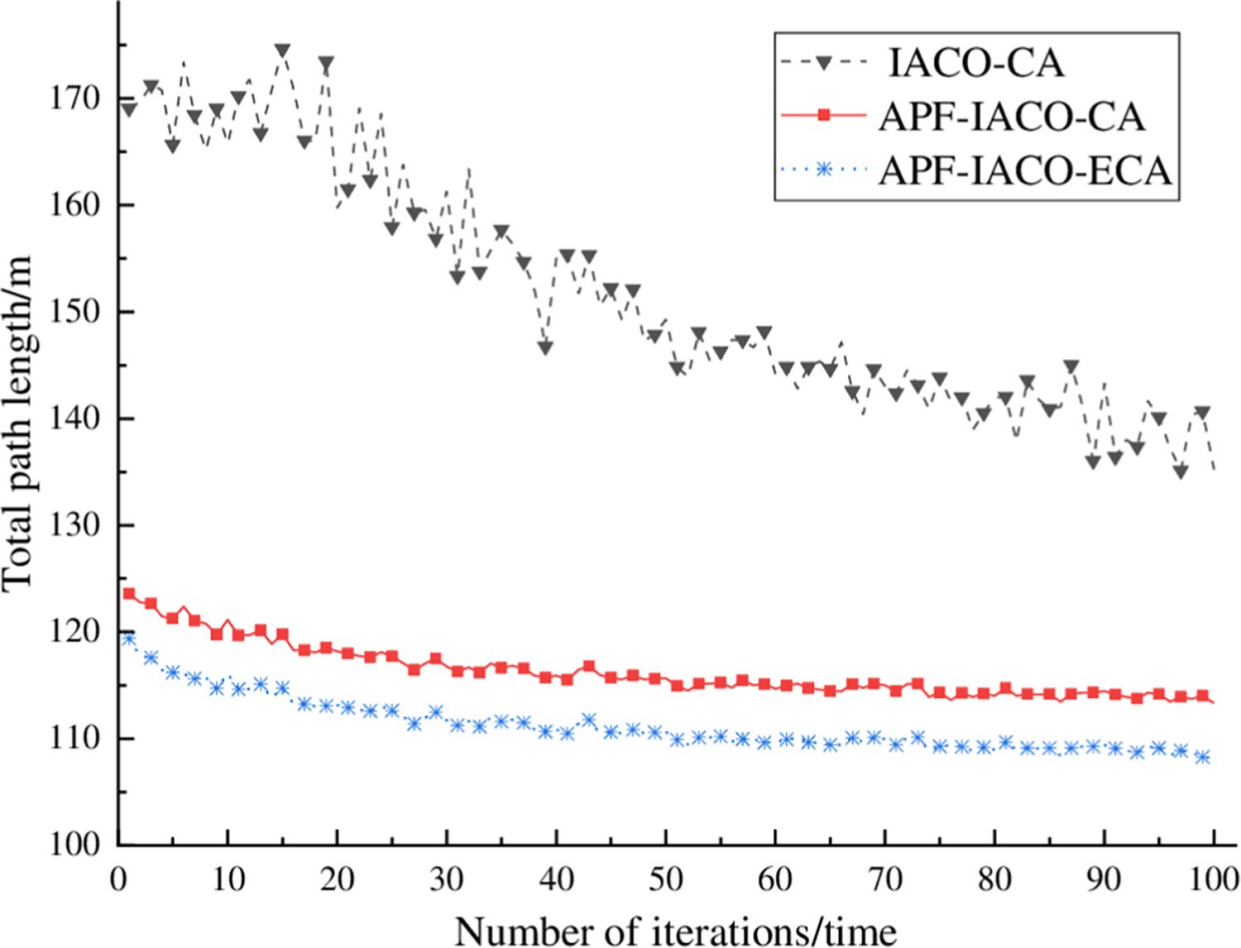
Dual exit planning total path length.

Fig 7 shows the curve of the total path length of a two-exit complete evacuation with iteration times. The total path length of Iaco-ca programming fluctuates greatly, but the overall trend of the total path length decreases with the number of iterations. The total path length of APF-IACO-CA and APF-IACO-ECA decreases with the iteration increase. As shown in Table 5, the shortest total path length of APF-IACO-CA is 113.33 m. The shortest total path length of APF-IACO-ECA is 108.15 m, and the total path length is shortened by 4.57%. The APF-IACO-ECA algorithm is more conducive to planning people’s paths to quickly reach the exit.

**Table 5:** Comparison of path distances.

algorithm	IACO-CA	APF-IACO-CA	APF-IACO-ECA
distance/m	135.26	113.33	108.15

#### 4.2.3. Planning total calculation time analysis

**Fig 8:**
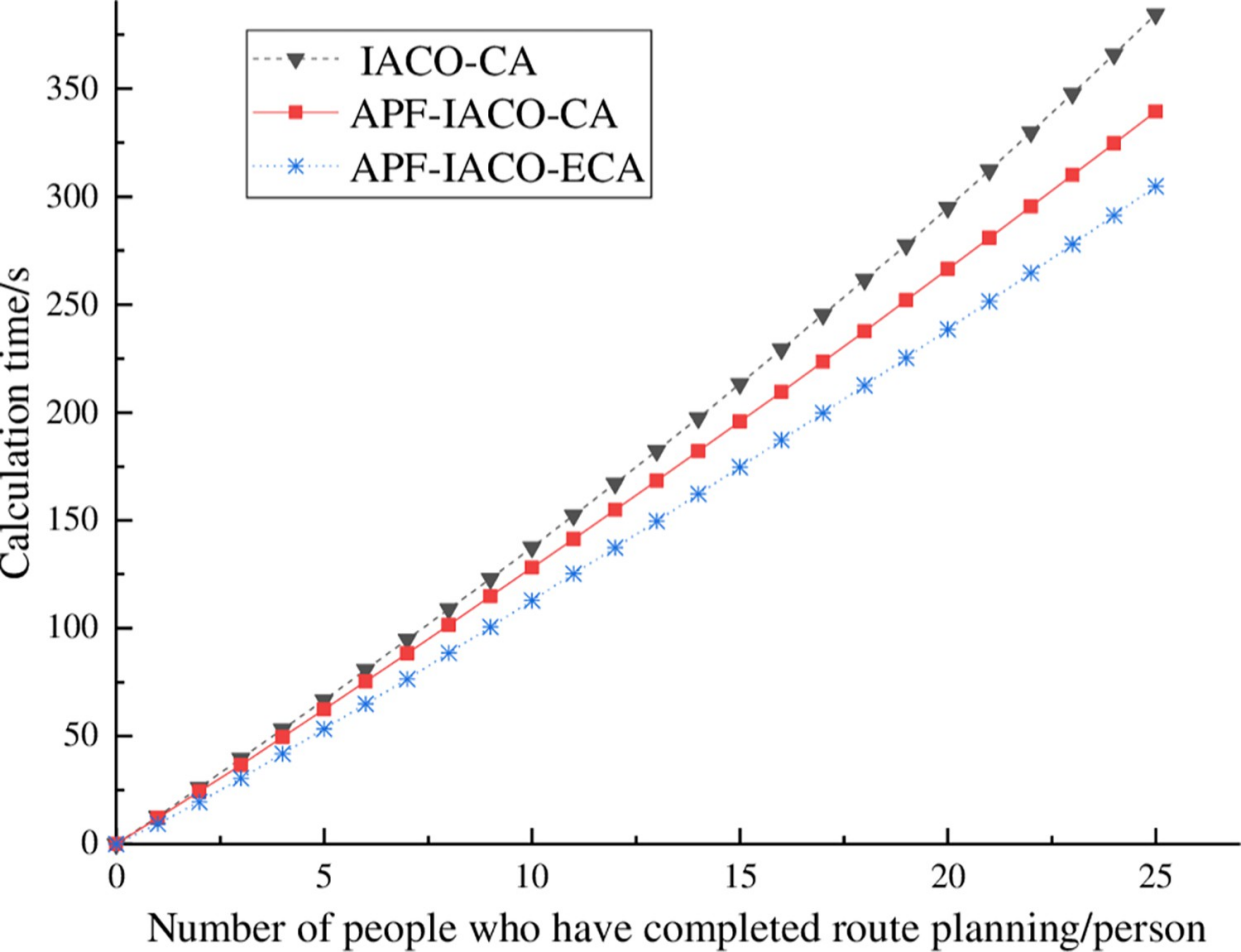
Total calculation time curves.

Fig 8 and Table 6 respectively show the curve and statistical table of the number of completers in the dual-exit scenario, which takes 384.38 s for IACO-CA, 339.38 s for APF-IACO-CA, and 304.78 s for APF-IACO-ECA. The APF-IACO-ECA algorithm has the fastest computation speed, reducing the computation time by 79.60 s and saving 20.71% compared with the IACO-CA algorithm and reducing the computation time by 34.6 s and saving 10.20% compared with the APF-IACO-CA algorithm. When the actual situation is combined with the path planning, the optimal path planning can be completed more effectively, proving the APF-IACO-ECA algorithm’s effectiveness.

**Table 6:** Comparison of calculation time.

algorithm	IACO-CA	APF-IACO-CA	APF-IACO-ECA
time/s	384.38	339.38	304.78

### 4.3 Partial scene simulation of a teaching building

**Fig 9:**
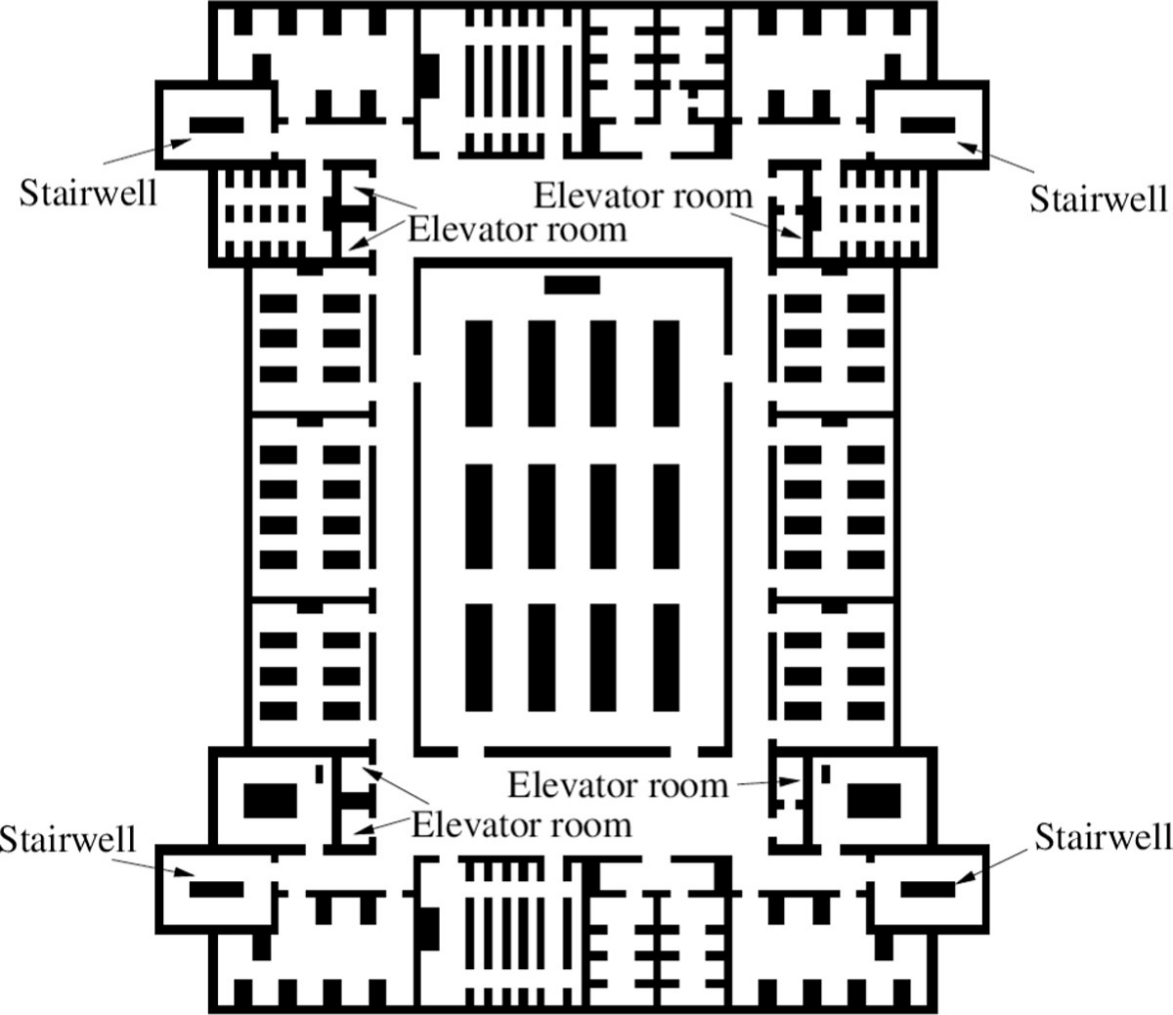
Cellular automata evacuation area model.

Some areas of a teaching building are taken as evacuation scenes to verify the effect of the algorithm in the actual scene. As shown in Fig 9, the maximum span of this evacuation area is 37.6 m in a transverse direction and 45.6 m in a longitudinal direction, and it covers an area of 1534m2. By the classroom, laboratory, office, conference room, toilet, and other space, 10 entrances and exits, including 6 elevators, Stairwell 4, personnel to reach the stairs or elevator evacuation is successful.

**Fig 10:**
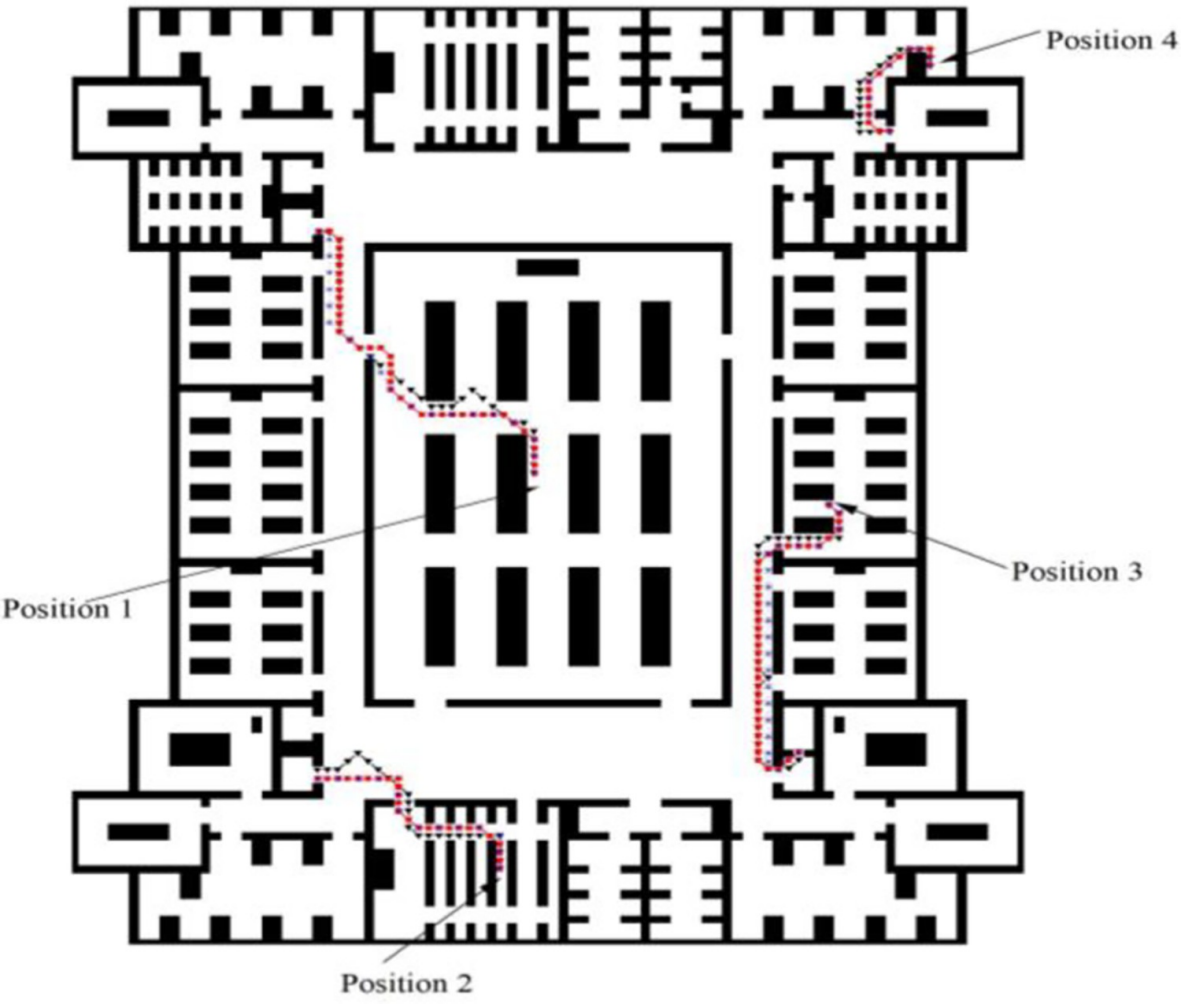
Path planning diagram.

Select four different initial positions, as shown in Fig 10.

#### 4.3.1. Path smoothness analysis

According to Table 7, the number of turns planned by IACO-CA is 33, and the number of turns is 129. The number of turns and planning times of APF-IACO-CA is 31 and 123, respectively, and the number of turns and planning times of APF-IACO-ECA are 27 and 64, respectively. The APF-IACO-ECA algorithm is the smoothest path planning algorithm in the actual scene, and it has the most advantages and is more applicable than other algorithms.

**Table 7:** Path comparison.

Algorithm	IACO-CA				APF-IACO-CA				APF-IACO-ECA			
Location	1	2	3	4	1	2	3	4	1	2	3	4
Number of turns/turns	9	7	12	5	10	6	9	6	7	7	8	5
Planning frequency/frequency	39	27	44	19	40	24	40	19	20	13	22	9

#### 4.3.2. Convergence effect analysis

As shown in Table 8, the path length of APF-IACO-CA converges to the minimum of 17.42m, 10.66m, 16.93 m, and 6.83m after 40,33,88 and 35 iterations, respectively After 33,28,29 and 27 iterations, the path length of APF-IACO-ECA converges to the minimum of 17.14m, 10.35m, 16.36m and 6.35m respectively. Compared with APF-IACO-ECA, the number of iterations of APF-IACO-ECA is reduced by 39.38%. The APF-IACO-ECA algorithm has the fastest convergence, the shortest path, the highest computation efficiency, and the best optimization effect.

**Table 8:** Shortest path convergence table.

Algorithm	APF-IACO-CA				APF-IACO-ECA			
Location	1	2	3	4	1	2	3	4
Shortest path/m	17.42	10.66	16.93	6.83	17.14	10.35	16.36	6.35
Iterations at convergence Number of iterations/time	40	30	88	35	33	28	29	27

## 5 Conclusion

1) The simulation results show that the proposed APF-IACO-ECA algorithm converges faster than the IACO-CA algorithm and APF-IACO-CA algorithm, and the algorithm has higher computational efficiency and fewer turns, effectively reducing the shortest path of invalid nodes and redundant turning points, improve the smoothness of the path, planning efficiency, and the best optimizing effect.

2) The CA and ECA model based on potential field ant colony is constructed, and the path planning of the ECA model is better than that of the CA model. In the evacuation area with a single exit, the total evacuation path of 25 people planned by the APF-IACO-ECA algorithm is 7.75% shorter than that planned by the APF-IACO-CA algorithm, and the total calculation time is reduced by 10.20%. In the two-exit evacuation area, the APF-IACO-ECA algorithm is 4.57% shorter than that of APF-IACO-CA, and the total calculation time is 10.20% less.

3) According to the evacuation scene in part of a teaching building, the shortest path to the exit in four different initial positions is planned using various algorithms. Compared with the APF-IACO-CA algorithm, the iterative times of the APF-IACO-ECA algorithm were reduced by 39.38%, and the number of iterations was reduced by 39.38%. The effectiveness of the APF-IACO-ECA algorithm is further verified in the actual scene.

## Supporting information

S1 Data (ZIP)
